# Inbreeding Avoidance by Recognition of Close Kin in the Pea Aphid, *Acyrthosiphon pisum*

**DOI:** 10.1673/031.012.3901

**Published:** 2012-03-23

**Authors:** Ming H. Huang, Marina C. Caillaud

**Affiliations:** ^1^ Department of Entomology, University of Arizona, Tuscon, Arizona, USA; ^2^ Department of Biology, Ithaca College, Ithaca, New York, USA

**Keywords:** Aphididae, cryptic female choice, inbreeding avoidance mechanisms, inbreeding depression, mating behavior

## Abstract

Inbreeding depression has detrimental effects on many organisms, but its effects are potentially greater in organisms that have at least one asexually reproducing life stage. Here, the existence of severe inbreeding depression upon selfing (r = 1) in the cyclic parthenogenetic aphid *Acyrthosiphon pisum* (Harris) (Hemiptera: Aphididae) is documented. Egg hatching success and offspring survival of inbred mating pairs are significantly lower than that of outbred mating pairs. Two possible mechanisms for avoiding selfing are examined: avoidance of partners of identical genetic makeup and avoidance of partners of the same body color (as a proxy for genetic similarity). Mating between males and females of the same color was as successful as mating between partners of different colors. In contrast, the success of mating between close kin was consistently reduced compared to that of mating between genetically unrelated partners. Interestingly, mating between close kin proceeded normally until the very last stage of the mating process. Thus, inbreeding avoidance appears to take place sometime between copulation and sperm transfer, suggesting that cryptic female choice may play a role in the process.

## Introduction

Inbreeding depression occurs when there is a decrease in fitness of offspring as a result of consistent breeding between closely related individuals (Charlesworth and Charlesworth 1987). The offspring of inbred individuals experience a decrease in fitness because of an increase in the occurrence of homozygous recessive alleles that are deleterious. Under most situations, the offspring that carry the homozygous deleterious alleles will not live past their early stages of development because of their inability to compete for limited resources or survive attacks by pathogens (Barrett and Charlesworth 1991). Offspring that survive to adulthood often face sterility, have sperm deformities, and are less able to court females (Pusey and Wolf 1996).

As a consequence, many organisms have evolved three main ways of avoiding inbreeding (Pusey and Wolf 1996). Townsend's voles and various birds avoid inbreeding by migrating away from natal environments to reduce the chances of mating with relatives (Pusey 1987; Lambin 1994). Other animals that exhibit sperm competition or cryptic female choice acquire multiple matings to ensure that some of the progeny will not be inbred (Rowley et al. 1993). A third mechanism used for avoiding inbreeding is to recognize and avoid mating with close kin (Pusey and Wolf 1996).

Organisms that reproduce both sexually and asexually within the same lifecycle (i.e., cyclic parthenogenesis) often face a very high level of inbreeding. Aphids are especially susceptible to inbreeding because approximately three quarters of their life cycle involves asexual reproduction (Dixon 1977). All offspring that are produced asexually are genetically identical to their parent except for males, which are almost identical since they have only one copy of the sex chromosome (males are XO). During the sexual part of the life cycle, all egg-laying females (i.e., oviparae) are wingless and males are either winged or wingless but are poor dispersers (Caillaud et al. 2002; Huang and Caillaud, personal observation). For non-host-alternating aphids such as *Sitobion avenae*, the fitness consequences of selfing are magnified because males and females of almost identical genomes can often be found on the same plant (Helden and Dixon 1997; Dedryver et al. 1998).

In the present study, mating between males and egg-laying females of the same genotype are studied in a non-host-alternating aphid species, the pea aphid *Acyrthosiphon pisum* (Harris) (Hemiptera: Sternorrhyncha). First, reproductive success in inbred mating pairs and outbred mating pairs is compared. Inbreeding depression upon selfing has been suspected (Via 1992) but not fully documented in this species. This study shows that selfing results in severe inbreeding depression in the pea aphid. Second, the question of whether pea aphids avoid inbreeding with close kin is examined. Mating attempts and actual copulation were unaffected by genetic relatedness. However, the success of mating between close kin appeared to be consistently reduced compared to that of mating between genetically unrelated partners, suggesting that female cryptic choice could play a role. Third, the possible role of body color in identification of genetically related individuals was studied. Body color could represent a non-specific recognition system, as well as a crude assessment of whether potential mates are different from self. It appears that pea aphids do not use the recognition of body color as a mechanism for avoiding inbreeding.

## Materials and Methods

All aphid genotypes considered in this study were collected in alfalfa fields (*Medicago sativa*) in five different locations in Tompkins County (New York, USA) in 1998 (except for genotype L9, which was collected in 1993). These genotypes have reproduced parthenogenetically (clonally) on alfalfa plants (cv. Oneida) since their collection.

To maintain parthenogenesis reproduction, colonies were kept at 20 °C with a 16:8 L:D photoperiod. To induce sexual reproduction, males and oviparae for each clone were induced separately in large 22 cm × 42 cm plastic containers containing several small pots of alfalfa, as described in Caillaud et al. (2002). On the first day of sexual induction, the length of day was decreased from 16 hours of light to 13 hours and 45 min. Daylight length was then gradually decreased in 15-min increments every other day until the day length decreased to 12 hours and 30 min. Each clone was then maintained at 15 °C and a 12.5:11.5 L:D photoperiod for five to six weeks. Only during the later part of this critical period were males and oviparae produced. After the production of sexual morphs was successfully induced, the production of males and oviparae persisted as long as the rearing conditions were kept constantly at 15 °C and 12.5 hours of daylight.

Males and oviparae of each clone were sorted into separate smaller cups of alfalfa once a large quantity of them started to appear (about six weeks after sexual induction started). Male and oviparae aphids can be easily recognized by the presence of two black claspers close to the tip of the abdomen (males) and thickened hind tibia (oviparae) (Miyazaki 1987). When sorting egg-laying females, it was important to collect them before they became adults to assure that they were virgins.

### Reproductive success of inbred mating

Two types of crosses were set up. First, males and oviparae of the same genotype were allowed to mate, thus generating inbred progeny (r = 1). Nine genotypes were subjected to selfing: L9, LSR1, LSR2, LSR3, PBR7, PBG7, PBR2, FVR1, and FVG1. Second, virgin oviparae from each of these genotypes were collected and mated with males of one of the other genotypes, thus generating outbred progeny: L9 × LSR1, LSR1 × LSR2, LSR2 × LSR3, LSR3 × FVG1, PBR7 × PBG7, PBG7 × LSR1, PBR2 × FVG1, FVR1 × LSR2, and FVG1 × PBR2.

Crosses were performed as described in Via (1992). All fertilized eggs produced throughout the life of the females were harvested, surface sterilized, and placed in an incubator under daily cycles of 4 °C during a 10 hour day and 0 °C during a 14 hour night. After about 100 days of this cold treatment, eggs were removed from the incubator and the hatchling progeny (fundatrices) were reared in Petri dishes containing alfalfa foliage until they reproduced.

Reproductive success was measured as (1) the percentage of eggs laid by females that successfully hatched into live fundatrices, and (2) the percentage of fundatrices hatched that matured into adults. For each variable, inbred and outbred mating pairs were compared using one-way ANOVA as implemented by the statistical package SPSS 10.0.

### Avoidance of inbreeding

The following genotypes were used for behavioral experiments: four red genotypes (LSR1, LSR2, LSR3, E125) and four green genotypes (FVG3, L9, LSG2, PBG7). Fully matured females ranging from six to 10 days old were used, since that is their most sexually active age, while mature males of any age were used, since the level of male sexual activity does not vary with age (Foret and Caillaud, unpublished observations). Experiments were always performed approximately four to seven hours after onset of daylight because pea aphids are most sexually active at that time of the day (Foret and Caillaud, unpublished observations).

The behavioral arena was designed to limit background stimuli which are known to disturb aphids. The base of the behavioral arena was 81 × 50 cm and the walls were 48 cm high. Each wall was covered with off-white paper. Four 10 × 10 cm pots, each containing four well-separated stems of alfalfa, were used in the experiments. Each stem of alfalfa was trimmed so that only three distinctly isolated sets of leaves remained. Each pot was placed in alternating forward and back positions to assure that the pots were not too close together. Doing this helped minimize the effects of activities in one pot influencing the activities in neighboring pots. A set of florescent lamps hanging over the experimental arena was used to facilitate the making of observations and to simulate daylight.

**Experiment #1: Do pea aphids avoid inbreeding by recognition of close kin?** The mating success of outbred and inbred mating pairs was compared. The mating pairs that were categorized as “inbred” consisted of oviparae and males that belonged to the same genotype (e.g., L9 × L9). We tested eight oviparae × male mating pairs: four outbred pairs (L9 × PBG7, LSR2 × LSR1, E125 × LSR3, FVG3 × L9) and four inbred pairs (PBG7 × PBG7, LSR1 × LSR1, LSR3 × LSR3, L9 × L9).

During each day of observation, each mating pair was assigned to one of the four available pots in the experimental arena. The positions assigned were rotated every observation day. This rotation helped minimize the unwanted effects of plant position on the results of the experiment. Approximately half an hour before the observations started, 12 virgin oviparae (three per mating pair) and 20 males (five per mating pair) were isolated into separate Petri dishes with a moistened piece of filter paper and one leaf of alfalfa. Oviparae were placed individually on each leaf of three of the four alfalfa stems in each pot. The egg-laying females were allowed five min to settle on the leaves before the males were released. Then, two to three males were placed on the tip of the stems of alfalfa and the time of introduction was noted. Hand-held stop watches were used to record the duration of various stages of the mating process. Since multiple mating can create inaccuracy in the results, oviparae were collected immediately after their first mating so that other males would not get a chance to mate with them. At the end of each observation day, the oviparae that mated were dissected (details below), and the ones that did not mate were discarded. The percentage of females successfully mated was calculated as the number of females from a particular mating pair that received sperm transfer divided by the total number of females used for that mating pair that day.

The mating success of outbred mating (i.e., E125 × LSR3, FVG3 × L9, L9 × PBG7, and LSR2 × LSR1) and inbred mating (i.e., L9 × L9, LSR1 × LSR1, LSR3 × LSR3, and PBG7 × PBG7) were compared using one-way ANOVA for each of the six variables measured (see Table 1 for variables measured) and the statistical package JMP 5.1.

**Experiment #2: Do pea aphids avoid inbreeding by recognition of body color?** Red and green pea aphid morphs often coexist within the same population (Markkula 1963). In addition, several aphid species have been shown to be attracted to the orange-yellow-green area of the color spectrum (Kring 1967). Although red lies outside the desired area of the spectrum, red aphid morphs could still be distinguished from green morphs because the body color of most red morphs often varies between a dull red to a pale yellow. The mating success of Intercolor vs. Intracolor mating pairs was compared. The mating pairs used were Green × Red (Intercolor), Red × Green (Intercolor), Green × Green (Intracolor), and Red × Red (Intracolor). Experiments were performed as described in Experiment #1. Males and oviparae were placed on the plants so that one pot had a Green × Green mating pair, a second pot had a Red × Red mating pair, a third pot had a Green × Red mating pair, and a fourth pot had a Red × Green mating pair.

The performance of Intercolor mating pairs was compared to that of Intracolor mating pairs also using one-way ANOVA for all variables and the statistical package JMP 5.1.

**Table 1.**
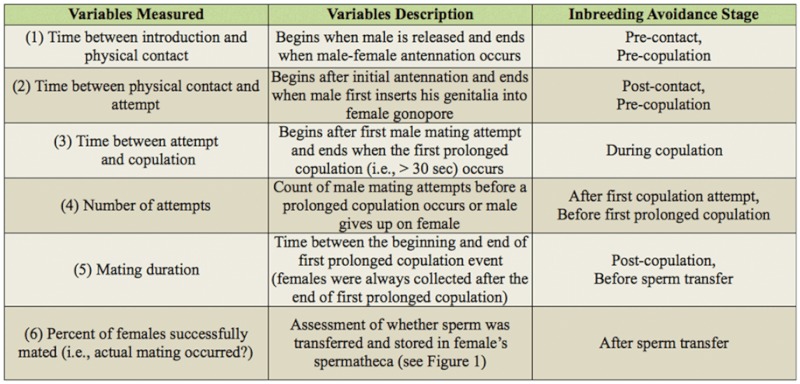
List of the variables measured in this study and the parameters used for each variable. Also shown is the stage where rejection of close kin can potentially occur if there is a significant difference in performance between inbred and outbred mating pairs for each variable.

### Dissection of mated oviparae and determination of actual mating

**Figure 1.**
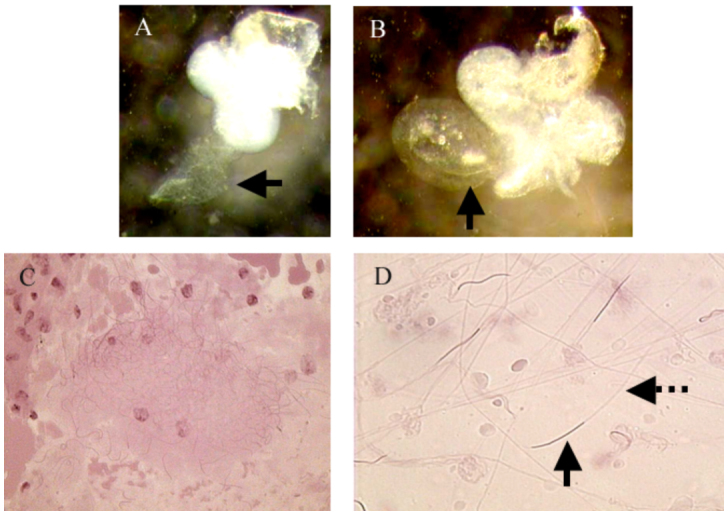
Two physical states of the spermatheca of a female pea aphid under 250× magnification (Ventral-lateral view): (A) empty flat spermatheca (solid arrow) of unmated female and (B) filled inflated spermatheca (solid arrow) of mated female. Shown under 400× magnification in (C) is the sperm bundle found inside an inflated spermatheca. Under 1000× magnification in (D), the elongated head (solid arrow) and long threadlike tail (broken arrow) of a single sperm can be seen. High quality figures are available online.

To determine whether or not sperm transfer had occurred, it was necessary to dissect each oviparae that had mated. Dissections were performed on a 2.54 cm × 7.62 cm glass slide at 160× magnification under a standard compound microscope to locate the spermatheca. When sperm transfer was not successful, the spermatheca of the oviparae was flat, transparent, and contained no sperm bundle (Figure 1A). However, if sperm transfer was successful, the spermatheca was well inflated and included a small bundle of opaque white material (Figure 1B). The staining of the internal material of the spermatheca using 10% aceto-orcein solution confirmed that the white mass was a bundle of spermatozoa (Figure 1C). Under 1000 × magnification, the elongated rod-like heads and the extremely long threadlike tails of individual spermatozoa can be seen (Figure 1D).

### Behavioral variables measured in experiments

Avoidance of inbreeding could potentially occur at any point in time between the search of a mate and after copulation occurs. If inbreeding avoidance occurs before any physical contact between the male and the oviparae, then pheromones perceived by the olfactory system, or visual cues perceived at a distance, may be the cues used by aphids to distinguish between genetically related and non-genetically related individuals. If inbreeding avoidance occurs only after copulation started, then cues perceived by the male and oviparae genitalia inside her reproductive tract may be mediating recognition of close kin. Many variables representing different stages of the mating process were measured in this study to identify the possible cues used by pea aphids to avoid inbreeding and the particular stage(s) at which rejection of close kin occurred (if at all) (see Table 1).

### Effect of oviparae weight on measured variables?

The difference between weights of males and oviparae could cause differences in mating success that are independent of the degree of genetic relatedness or body color. This factor was controlled for by the random selection of both oviparae and males for experiments. However, there was greater potential for female body weight to affect our results since oviparae had a greater variation in size. This possible effect was investigated further by weighing each of the egg-laying females that mated during observations using a Cahn Instruments Inc. C-31 Microbalance (accurate up to the thousandth milligram). Oviparae from a total of seven different female clones were weighed. A one-way ANOVA analysis using the statistical package JMP 5.1 showed that there was significant difference in oviparae weight across all clones used (*F*_6,98_ = 7.69,*p* < 0.01, *R*^2^ = 0.32). However, there was no significant relationship between oviparae weight and any of the six variables tested: (1) Time Between Introduction and Physical Contact (*t*_104_ = 0.65, *p* = 0.518, *R*^2^ = 0.004100), (2) Time Between Physical Contact and Attempt (*t*70 = 0.03, *p* = 0.974, *R*^2^ = 0.000016), (3) Time Between Attempt and Copulation (*t*_79_ = 0.26, *p*= 0.798, *R*^2^ = 0.000850), (4) Number of Attempts (τ = 0.015, *p* = 0.864, N = 79), (5) Mating Duration (*t*_103_ = -0.45, *p* = 0.654, *R*^2^ = 0.001970), and (6) Number of Actual Successful Matings (*χ*^2^ = 1.29, *p* = 0.256, *R*^2^ = 0.011000, N = 104). A regression analysis was used for variables (1), (2), (3), and (5); a log transformation was performed on variables (2) and (3) since the data points were not spread evenly in their respective residual plots. Since more than half of the males only made one attempt during variable (4), the data remained non-parametric even after transformation. Thus, a Kendall's Tau test (non-parametric regression) was used to test for a correlation between female weight and number of attempts. A logistic regression test was used for variable (6).

## Results

### Reproductive success of inbred mating

**Figure 2.**
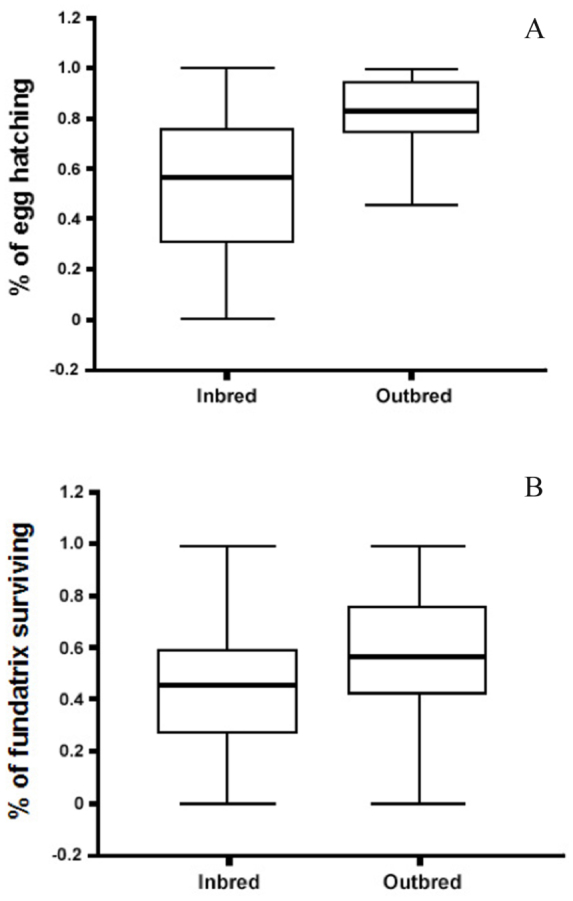
(A) Hatching success (percentage of eggs laid that hatched into live fundatrices) and (B) fundatrix survival (percentage of fundatrices that survived to adulthood) of inbred and outbred mating pairs. For each variable the mean, quartiles, and extreme values are shown. High quality figures are available online.

Eggs were collected in batches of 20 to 30 eggs. There were 77 batches of outbred eggs (total of 1925 eggs) and 73 batches of inbred eggs (total of 1752 eggs). The percentage of egg hatching of inbred crosses was significantly lower than that of outbred crosses (*F*_1,148_ = 45.7, *p* < 0.01, *R*^2^ = 0.236) (Figure 2A). The average hatching success for inbred crosses was 54% (SD = 10.1, N = 73), while that of outbred crosses was 81% (SD = 9.04, N = 77). A few genotypes subjected to inbreeding produced no fundatrices (hatching success of 0%) because all the eggs produced failed to hatch (i.e., PBG7 and LSR3).

The percentage of fundatrices that survived until adulthood of inbred crosses was also significantly lower then the one of outbred crosses (*F*_1,148_ = 13.6, *p* < 0.01, *R*^2^ = 0.084) (Figure 2B). The average fundatrix survival for inbred crosses was 43% (SD = 8.9, N = 73) while that of outbred crosses was 58% (SD = 7.2, N = 77).

### Avoidance of inbreeding

**Figure 3.**
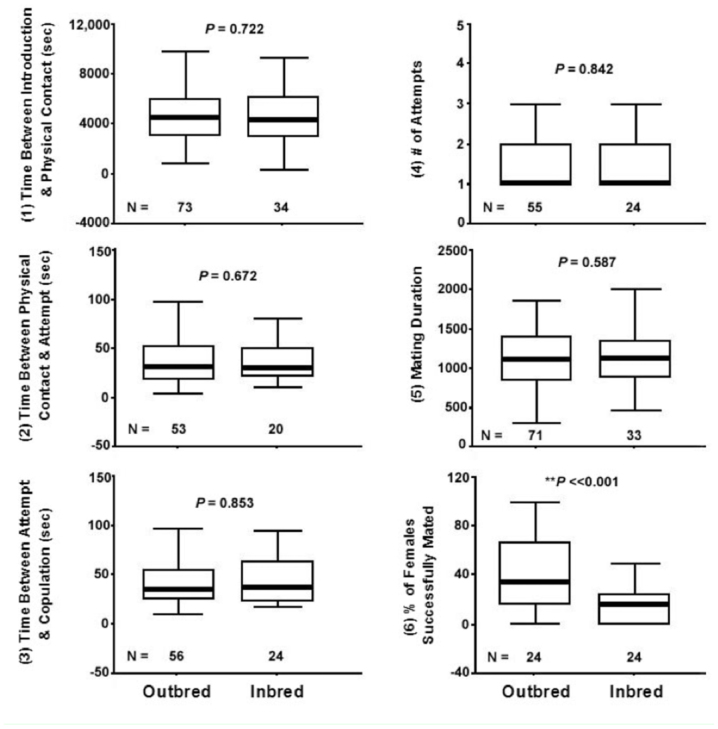
Mating success of outbred mating pairs versus inbred mating pairs: (1) Time Between Introduction and Physical Contact, (2) Time Between Physical Contact and Attempt, (3) Time Between Attempt and Copulation, (4) Number of Attempts, (5) Mating Duration, and (6) Percentage of Females Successfully Mated. For each variable the median, quartiles, and extreme values are shown. High quality figures are available online.

**Experiment #1: Do pea aphids avoid inbreeding by recognition of close kin?** Figure 3 shows the mating success of outbred and inbred mating pairs at various stages of the mating sequence. The performance of the two mating pair types was not significantly different for the Time Between Introduction and Physical Contact (*F*_1,105_ = 0.127, *p* = 0.723, *R*^2^ = 0.0012), Time Between Physical Contact and Attempt (*F*_1,71_ = 0.181, *p* = 0.672, *R*^2^ = 0.0025), Time Between Attempt and Copulation (*F*_1,78_ = 0.035, *p* = 0.853, *R*^2^ = 0.0004), Number of Attempts (*F*_1,77_ = 0.040,*p* = 0.842, *R*^2^ = 0.0005), and Mating Duration (*F*_1,102_ = 0.298, *p* = 0.587, *R*^2^ = 0.0029). However, the outbreds had a significantly higher Percentage of Females Successfully Mated than the inbreds (*F*_1,46_ = 15.9, *p* < 0.01, *R*^2^ = 0.2600). The outbreds had a mean of 40.28% (SE = 4.55, N = 24) while the inbreds had a mean of only 14.58% (SE = 4.55, N = 24).

**Figure 4.**
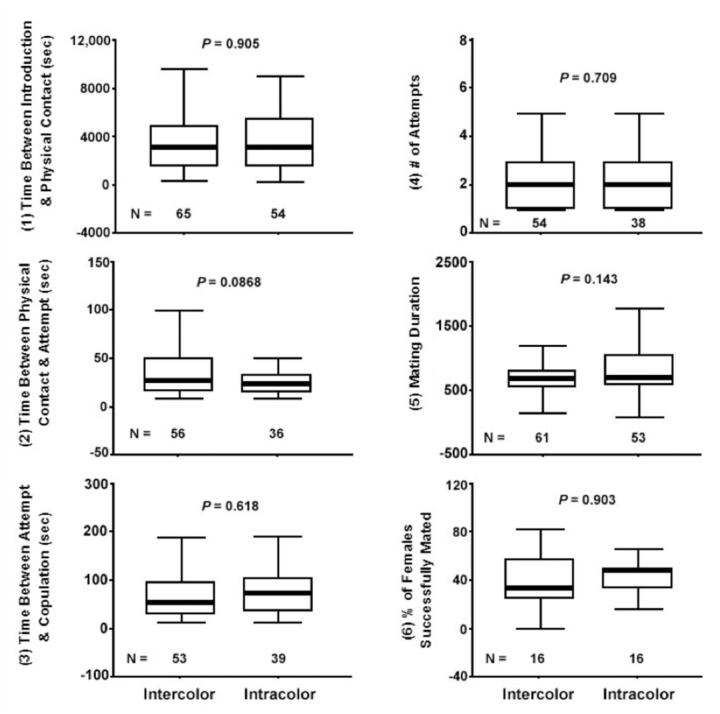
Mating success of Intercolor mating pairs versus Intracolor mating pairs: (1) Time Between Introduction and Physical Contact, (2) Time Between Physical Contact and Attempt, (3) Time Between Attempt and Copulation, (4) Number of Attempts, (5) Mating Duration, and (6) Percentage of Females Successfully Mated. For each variable the median, quartiles, and extreme values are shown. High quality figures are available online.

**Experiment #2: Do pea aphids avoid inbreeding by recognition of body color?** Figure 4 shows the mating success of Intercolor mating pairs (i.e., Green × Red and Red × Green) and Intracolor mating pairs (i.e., Green × Green and Red × Red). The performance of Intercolor pairs was not significantly different than that of Intracolor pairs in either Time Between Introduction and Physical Contact (*F*_1,117_ = 0.010,*p* = 0.905, *R*^2^ = 0.00012), Time Between Physical Contact and Attempt (*F*_1,90_ = 3.00, *p* = 0.0868, *R^2^* = 0.03200), Time Between Attempt and Copulation (*F*1,90 = 0.250, *p* = 0.618, *R*^2^ = 0.00280), Number of Attempts (*F*_1,90_ = 0.140, *p* = 0.709, *R*^2^ = 0.00160), Mating Duration (*F*_1,112_ = 2.18, *p* = 0.143, *R*^2^ = 0.01900), or Percentage of Females Successfully Mated (*F*_1,30_ = 0.015, *p* = 0.903, *R*^2^ = 0.00051).

## Discussion

**Figure 5.**
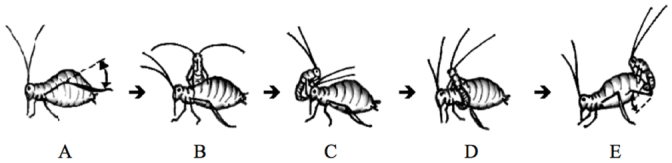
Mating sequence of pea aphids: (A) female signals to males by releasing pheromones from her hind tibia, (B) male gets a firm grip on the side of a female, (C) - (D) male makes 1 – 3 full circles around female, tapping her body with the tip of his abdomen as he encircles her, (E) female lifts up her abdomen while the male places his genitalia into the female's gonopore and continuously strokes the side of the female's abdomen with his hind legs. High quality figures are available online.

**Figure 6.**
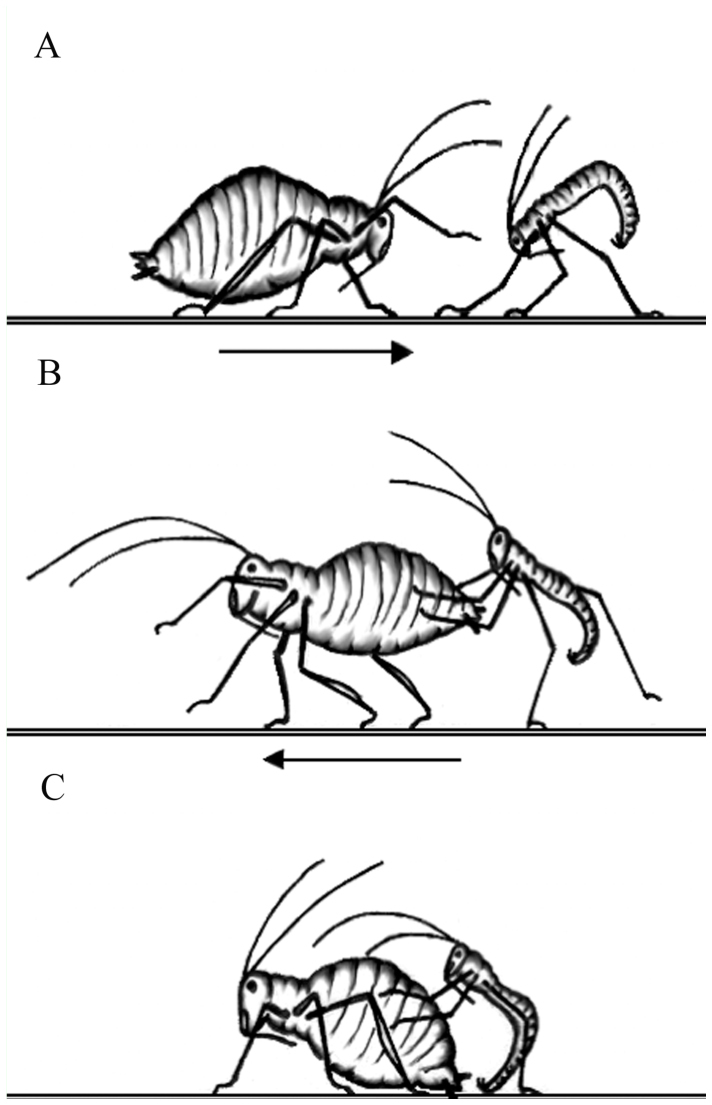
Methods that pea aphid females use to prevent males from mating successfully: (A) female lunges forward at the male, pushing him with her forelegs, (B) female walks away from the advancing male, while male tries to hold on to her, (C) female holds her abdomen flat against the substrate, preventing the male from copulating with her. High quality figures are available online.

The main objective of this study was to test the hypothesis that pea aphids avoid selfing. To examine inbreeding avoidance in pea aphids, their general mating behavior was first characterized. Observations revealed that females initiate the mating process by waiving one of their hind tibia in an up-and-down motion, releasing sex pheromones (Pickett et al. 1992) (Figure 5A). Within one to two minutes, searching males would turn in the direction of the females and run towards her at a rapid speed. When the male reached the female, mutual antennation occurs. If the male was successful at courting the female, he would begin tapping her body with the tip of his abdomen while circling around her body (Figures 5B, 5C, 5D). The male then would insert his genitalia into the female's gonopore while continuously stroking the lateral side of her abdomen for approximately 10–20 seconds in an up-and-down motion (Figure 5E). If the female was not receptive of the male, she would exhibit one or more of the three following behaviors: turn around and lunge forward at the male (Figure 6A); start walking forward while the male tries to hold on (Figure 6B); and/or hold her abdomen flush against the substrate to prevent copulation (Figure 6C). These observations suggest that the female pea aphid is most likely the choosy sex. Also, the constant interaction between females and males throughout the mating process suggests that the recognition of certain cues during one or more phases may aid the avoidance of mating with close kin.

After revealing that inbred matings indeed result in lower fitness in pea aphids (Figure 2), we tested whether pea aphids avoided inbreeding by recognizing close kin (same genotype) or by responding to the body colors of surrounding individuals. Responding to body color would be less precise than responding to clone-specific cues, but recognizing body color could potentially help detect closely related individuals from far distances because the assessment of visual stimuli would not require physical contact. In contrast, the recognition of identical clones would be more precise, but would require the acquisition of close-range cues such as olfactory chemoreception, contact chemoreception, or internal cues.

Six variables in the pea aphid mating process where recognition of genetically related individuals could occur were measured: (1) time between introduction and physical contact, (2) time between physical contact and attempt, (3) time between attempt and copulation, (4) number of attempts, (5) mating duration, and (6) percentage of females successfully mated. Inbred and outbred mating pairs did not differ in variable (1) (Figure 3). Almost all males reacted relatively quickly after oviparae performed the leg-waving behavior seen in Figure 5A. This shows that long-range sexual pheromones emitted by oviparae are not involved in inbreeding avoidance. Instead, this long-range signal is most likely used only for recognition at the species level. Studies by Steffan (1990) on two other aphid species, *Sitobion avenae* and *Dysaphis plantaginea*, showed similarly strong responses by males to pheromone released by oviparae of the same species before making physical contact. The fact that inbred and outbred mating pairs did not differ in variables (2) - (5) shows that inbreeding avoidance does not occur before a prolonged copulation event. This suggests that cues perceived at a close range (e.g., epicuticular hydrocarbons) are not used to assess the genetic relatedness of individuals. Instead, inbred and outbred mating pairs differ only in variable (6) (Figure 3), suggesting that internal pre or post-copulatory cues during physical coupling of the male and oviparae genitalia allow recognition of close kin or self clones by males, oviparae, or both sexes. In contrast, Intracolor and Intercolor mating pairs did not exhibit significant differences in any of the variables measured (Figure 4). These results show that body color is not used as an indirect indication of genetic relatedness.

So, what may be occurring during the physical coupling between inbred partners? Female cryptic choice involves the manipulation of sperm after it is transferred to the spermatheca, which affects the sperm's chances of surviving long enough to fertilize an egg (Eberhard 1996). An example of female cryptic choice is the selective movement of stored sperm to specific areas of the spermatheca, which causes biased sperm usage (e.g., *Chorthippus parallelus* (Orthoptera) (Bella et al. 1992)). In the case of the pea aphid, two pieces of evidence suggest that oviparae rather than males are in control of sperm transfer during the copulation stage, and that a form of cryptic female choice is involved. First, dissections show that the eggs of oviparae are many magnitudes larger in size than male spermatozoa. Since larger gametes require so much more energy to produce, it would be more costly for oviparae than males to allow self-inbred fertilizations to occur. Second, males mated as long with inbred oviparae as with outbred oviparae (∼ 20 min), yet many that achieved a prolonged copulation event with inbred oviparae and were in the right position for transferring sperm did not successfully transfer sperm, or sperm was transferred but not taken into the spermatheca. In fact, some copulations that lasted up to 40.5 minutes did not result in successful sperm transfer. This suggests that males remained interested in transferring sperm throughout copulation but the oviparae had some method of preventing their sperm from reaching their spermatheca, yet still allow the male to go through with the copulation. Allowing unfavorable males to go through with the motions of copulation can be advantageous for the oviparae because it may prevent constant male harassment. One possible mechanism used by females to prevent sperm transfer is to make it difficult for males to deliver sperm by physically manipulating her internal genitalia. A similar mechanism has been described in the golden orb-weaving spider (Christenson 1990). This study revealed that the oviduct of this female spider species hardens after each progressive mating, which makes copulations with each successive male more and more irregular. Another possible mechanism would be a quick expulsion of delivered sperm immediately after copulation is completed, as documented in the fly *Dryomyza anilis* (Otronen 1990).

Recognition of genetically identical individuals using very specific cues within the oviparae during copulation may be the most suitable mechanism for pea aphids. Recognizing different body colors to avoid inbreeding is not very effective because it gives information that is too vague; individuals of the same body color can either be a genetically identical clone, close relative, or non-relative. Since mating with an identical clone has greater detrimental effects (Figure 2), recognizing individuals that are exact clones (r = 1) will be more important than recognizing individuals that are just close relatives (r < 1) and those that are nonrelatives (r « 1).

An alternative mechanism commonly used by mammals and birds for inbreeding avoidance is dispersal. However, this mechanism is unlikely to be effective in pea aphids. As mentioned in the introduction, pea aphid oviparae are always wingless and relatively slow walkers. Therefore, it would be very difficult for them to travel very far from natal habitats without being preyed upon or parasitized. Oviparae also cannot travel long distances for long periods of time because they constantly need to feed in order to obtain enough nourishment to produce eggs and survive. Given the short period for sexual reproduction, the pea aphid oviparae would not have enough time to disperse a short distance, feed, and then disperse further. Although some males do have wings (Caillaud et al. 2002), they are not the most diligent or effective flyers (Huang and Caillaud, personal observation). Most winged individuals can only fly upward in an awkward swirling motion. As a result, winged pea aphids would have to rely heavily on strong winds to move them forward. Since transportation via the wind in nature is not very reliable and consistent, it would be very difficult for winged individuals to make much progress in the desired direction. As a result, it is highly unlikely that pea aphids would rely on dispersal via flight as a way of avoiding inbreeding.

Another possible mechanism of avoiding inbreeding not examined in this study is the use of extra-pair matings to neutralize the effects of inbreeding (Tregenza and Wedell 2002). A study by Foret and Caillaud (unpublished observations) shows that pea aphid males mate, on average, with six different oviparae in a six-hour window; one oviparae even mated with eight different males. However, since pea aphids reproduce asexually throughout most of their lifecycle and all morphs are poor flyers, it is expected that natural populations of pea aphids would consist mostly of individuals that are of the same exact clone. Therefore, the probability of encountering an individual with a different genetic makeup by chance is relatively low. As a result, acquiring multiple matings alone would not be effective enough for pea aphids to consistently avoid inbreeding.

## Conclusion

This study revealed that pea aphids could avoid breeding with close kin. Avoidance of selfing only occurred during the physical coupling of the oviparae and male genitalia or after copulation. Cryptic female choice may be used to eliminate sperm from males that are too closely related. Despite the pea aphid's ability to reduce inbreeding by recognition of close kin and self clones at the post-copulatory stage, about 14.6% of the inbred mating pairs still resulted in successful transfer of sperm (Figure 3). It remains to be seen whether additional mechanisms intervene after sperm is transferred to the spermatheca but before the egg is fertilized, which further reduces the chance of creating inbred progeny. After sperm enters the spermatheca, two possible events may prevent the egg's fertilization. Stored sperm can either be eliminated through sperm competition or further cryptic female choice. Sperm competition is the elimination of one male's sperm by another male (Smith 1984). For example, males can stimulate females to empty the sperm deposited by a previous male by rubbing the female's oviduct with his genitalia (e.g., *Metaplastes ornatus* (Orthoptera) (von Helversen and von Helversen 1991)). The male's rubbing behavior presumably mimics the movement of eggs through the oviduct during fertilization.

Males can also physically plug the cavity of a female's external genitalia with a mass of material after insemination occurs (e.g., Funnel-web spider (Masumoto 1993)). Sperm competition may occur in pea aphids since there is multiple mating in this species (Foret and Caillaud, unpublished observations). However, sperm competition has never been documented in pea aphids. Future experiments using pea aphid microsatellite markers (e.g., Caillaud et al. 2004) for assessing paternity need to be performed to examine the respective role of cryptic female choice and sperm competition in inbreeding avoidance.
